# A rare presentation of Austrian syndrome with septic arthritis in an immunocompetent female

**DOI:** 10.1186/s43044-019-0010-6

**Published:** 2019-09-09

**Authors:** Ayman Battisha, Bader Madoukh, Ahmed Altibi, Omar Sheikh

**Affiliations:** 1 University of Massachusetts Medical School—Baystate, 759 chestnut street, Springfield, MA 01199 USA; 2 Overland Park Regional Medical Center-HCA Midwest Health, Overland Park, USA; 3 Henry Ford Health System, Detroit, USA; 4 University of Texas Health Science Center at San Antonio, San Antonio, USA

## Abstract

**Background:**

Austrian syndrome, which is also known as Osler’s triad, is a rare aggressive pathology consisting of pneumonia, endocarditis, and meningitis caused by *Streptococcus pneumoniae* and carries drastic complications.

**Case presentation:**

A case of a 68-year-old female with a past medical history of hypertension and had a recent viral influenza is presented. She developed bacterial pneumonia, endocarditis with mitral and aortic vegetations and perforation, meningitis, and right sternoclavicular septic arthritis. Two prior case reports have described sternoclavicular septic arthritis as part of Austrian syndrome. Our case is the third case; however, it is the first case to have this tetrad in an immunocompetent patient with no risk factors, i.e., males, chronic alcoholism, immunosuppression, and splenectomy.

**Conclusions:**

Clinicians should maintain a high index of suspicion for the possibility of sternoclavicular joint septic arthritis as a complication of Austrian syndrome in immunocompetent patients.

## Background

*Streptococcus pneumoniae*, a gram-positive diplococcus, is the most common organism seen in patients with community-acquired pneumonia and hospital-acquired pneumonia as well as bacterial meningitis. The prevalence of all *S. pneumoniae* infections decreased after the introduction of penicillin in 1928 [1]. Additionally, the introduction of pneumococcal vaccination in 1977 further reduced the incidence of infection via primary prevention in the USA [2]. *Streptococcus pneumoniae* can rarely present in a triad of endocarditis, meningitis, and pneumonia, known as Osler’s triad, named after the Canadian Physician Sir William Osler who first described it in 1881. Osler wrote, “Meningitis is a very rare complication of pneumonia and may occur apart from endocarditis” [3]. Today, this triad is known as Austrian syndrome after Dr Robert Austrian, who was the first one to describe it in paper and publish it in 1957 [4]. Despite detection and treatment of the disease, contemporary cases have been reported in the literature. These cases tend to occur in populations that have predisposing risk factors, such as chronic alcoholism, male sex, immunosuppression, and splenectomy [5]. A literature review revealed that 54 cases of Austrian syndrome have been reported. Moreover, untreated pneumococcal infections can rarely progress to a disseminated stage and lead to a sternoclavicular septic arthritis [6]. Austrian syndrome with sternoclavicular septic arthritis has been described only twice in the literature in an HIV-positive male and immunocompromised male, respectively [7]. Our case is the second case to describe this tetrad of pneumonia, meningitis, endocarditis, and sternoclavicular septic arthritis. What makes our case more atypical is that it was a female patient, immunocompetent, with no other risk factors of Austrian syndrome that have been described before.

## Case presentation

We present the case of a 68-year-old female with a recent viral influenza who presented to the emergency department with altered mental status. Around 7 days prior to admission, she started developing a productive cough associated with body aches and fatigue. She then started getting confused and that was when she was brought to the hospital. She did not have a significant medical history except for hypertension that was controlled well. Her only medication was amlodipine.

In the ED, she was found to have a high-grade fever of 100.8 °F, tachycardia (pulse 115), tachypnea (respiratory rate 30/min), and normal blood pressure of 130/70. Her Glasgow Coma Scale was 13. She was found to have left basilar crackles, pan-systolic murmur that is louder at the apex, and weakness in the right-sided upper and lower extremities.

**Fig. 1:**
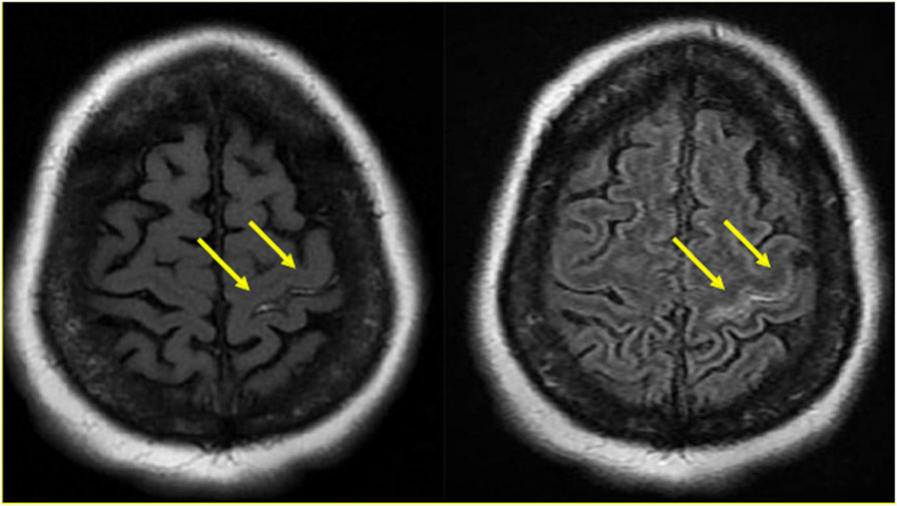
MRI of the brain demonstrating meningeal enhancement from pneumococcal dissemination

**Fig. 2:**
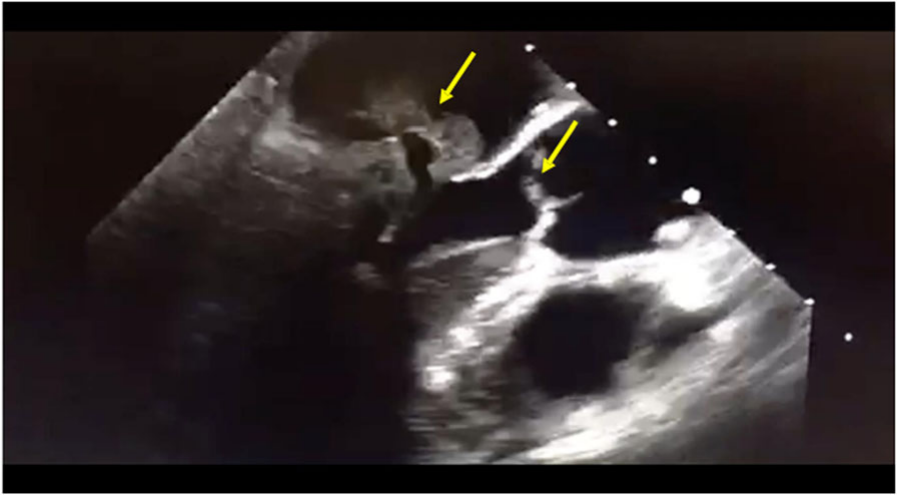
Transesophageal echocardiography (TEE) demonstrating mitral and aortic valvular vegetations

Chest X-ray showed multifocal infiltrates suggestive of pneumonia. CT of the head was unremarkable. Brain MRI demonstrated leptomeningeal enhancement suggestive of meningitis or septic emboli from the heart (Fig. 1). CSF analysis showed leukocytosis (WBC 2345/mm3, 83% neutrophils), high protein, low glucose, and positive *Streptococcus pneumoniae* PCR. Sputum and blood cultures grew *Streptococcus pneumoniae* as well. Her hemoglobin was 11, platelets 20,000 per mm^3^, ESR 60 mm/h, white blood cells 18,000 per mm^3^, C-reactive protein 447 mg/liter, and random blood glucose 6.4 mmol/liter. Initial transthoracic echocardiogram was negative for endocarditis. Due to a high index of suspicion for endocarditis, a transesophageal echocardiography was performed and revealed multiple echodensities on both mitral valve leaflets, the largest measuring 3 × 1 cm. Also, there was a perforation of the mid portion of anterior mitral valve leaflet and sever regurgitation. In addition, a smaller echodensity measuring 0.4 cm in diameter was noted on the aortic valve at the commissure between the left and noncoronary cusps (Fig. 2). Further clinical assessment and workup also revealed septic right sternoclavicular (SC) joint.

She was initially given vancomycin (20 mg/Kg IV q 8 h) and piperacillin-tazobactam (4.5 g IV q 6 h), which was subsequently narrowed to ceftriaxone (2 g IV q 12 h) following the culture and sensitivity results. She had a prolonged hospital course that required intravenous antibiotics, drainage of the right septic SC joint effusion, surgical mitral valve replacement, removal of the vegetations, and replacement of the mitral valve by a mechanical prosthetic valve was performed. During the postoperative period, the vital signs remained stable with no high fever. However, acute renal failure developed in the second week of the postoperative period. Nephrotoxic agents were discontinued. The patient was treated by hemodialysis until the creatinine levels returned to normal levels. The treatment was completed in 6 weeks, and she was discharged with a dramatic clinical improvement towards the end of her hospital stay. She received rehabilitation therapy after discharge from the hospital.

## Discussion

According to Patadia et al., risk factors classically associated with development of Austrian syndrome include immunocompromised status, male sex, chronic alcoholism, dural fistulas, and splenectomy [5]. Although our patient had a preceding viral pneumonia, which likely placed her at a risk for superimposed bacterial pneumonia, none of the abovementioned risk factors were found in our patient. The sequence of infection occurrence starting in the lungs followed by dissemination to extrapulmonary sites likely overwhelmed her immune system. In result, seeding in the sternoclavicular joint space occurred in the setting of bacteremia. According to Shirtliff and Mader, there are three mechanisms of seeding in an SC joint: direct spread of bacteria from an adjacent focus of infection, hematogenous dissemination, and trauma [8], the second of which fits in the case of this patient.

Austrian syndrome complicated by SC joint infection has been reported twice in the literature [7].

An article by Bindroo et al. presented the case of a 60-year-old male, who had known predisposing factors in his medical history, with Austrian syndrome and secondary SC joint infection [9]. However, there were a couple of differences between the two cases: our patient was a female, 68 years of age, and sensitive to penicillin, whereas the patient in the Bindroo et al. article was a male, 60 years old, and resistant to a 6-week course of ceftriaxone [9]. Our case is the first in the literature to present with Austrian syndrome complicated by SC joint septic arthritis in an immunocompetent female with hypertension as her only comorbidity. This case uniquely challenges the common perception that Austrian syndrome occurs in older, alcoholic males. As an immunocompetent female, it is not known whether the syndrome manifested as it classically would have developed in the pre-penicillin era because penicillin was discovered in 1928, and Austrian syndrome was clinically characterized in 1957 [1].

A different study by Sewlall et al. presents a case of an HIV patient afflicted with Austrian syndrome complicated by septic arthritis as a result of “invasive pneumococcal infection” (IPI) [7]. The case was another example of Austrian syndrome that occurred in the presence of underlying risk factors, including male sex and immunocompromised status [7]. Perhaps immunomodulation may not necessarily serve as a defense mechanism against pneumococcal dissemination. However, it may facilitate recovery from Austrian syndrome upon early antibiotic administration, as demonstrated by our patient’s hospital course. The class of antibiotic differed between Sewlall’s patient and our patient because of penicillin resistance [7].

One limitation of the preceding studies, including our case, is the lack of generalizability of results to future cases of Austrian syndrome. The reason is that the timing of antibiotic administration relative to disease onset likely varied from case to case. One case of Austrian syndrome may have progressed more than another case prior to receiving antibiotics. This variability makes it difficult to determine whether disease outcomes are consistently modified as a result of early antibiotic administration. However, earlier administration of antibiotics would logically contribute to a more favorable patient outcome.

## Conclusion

In light of our case presentation and the current literature about Austrian syndrome, clinicians should maintain a high index of suspicion for the possibility of sternoclavicular joint septic arthritis as a complication of Austrian syndrome in immunocompetent patients with pneumococcal endocarditis irrespective of antibiotic sensitivity, for earlier detection, prompt treatment, and better patient outcomes.

## Data Availability

Yes
